# Microstructure and Hardness Characteristics of Swing-Arc SAW Hardfacing Layers

**DOI:** 10.3390/ma17102310

**Published:** 2024-05-13

**Authors:** Zhengyu Zhu, Maoyang Ran, Xuyang Li, Pichang Ma, Shubin Liu, Jiayou Wang

**Affiliations:** 1 Provincial Key Lab of Advanced Welding Technology, School of Materials Science and Engineering, Jiangsu University of Science and Technology, 666 Changhui Road, Zhenjiang 212100, China; zzy_just@163.com (Z.Z.); 221210601516@stu.just.edu.cn (M.R.); 17315764033@163.com (X.L.); pichang2024@163.com (P.M.); 2 Jiangsu Province Marine Equipment Intelligent Engineering Technology Research and Development Center, Jiangsu Maritime Institute, 309 Gezhi Road, Nanjing 211170, China; 3 Xuzhou Construction Machinery Technician College, 26 Zhujiang Road, High-Tech Zone, Xuzhou 221116, China

**Keywords:** swing-arc submerged arc welding, hardfacing layers, weld formation, microstructure, hardness uniformity

## Abstract

Hot-rolled backup rolls are widely used in steel rolling and usually need to be repaired by arc hardfacing after becoming worn. However, a corrugated-groove defect commonly occurs on the roll surface due to the uneven hardness distribution in the hardfacing layers, affecting the proper usage of the roll. Accordingly, a new swing-arc submerged arc welding (SA-SAW) process is proposed to attempt to solve this drawback. The microstructure and hardness are then investigated experimentally for both SAW and SA-SAW hardfacing layers. It is revealed that a self-tempering effect occurs in the welding pass bottom and the welding pass side neighboring the former pass for both processes, refining the grain in the two areas. In all the zones, including the self-tempering zone (STZ), heat-affected zone (HAZ), and not-heat-affected zone in the welding pass, both SAW and SA-SAW passes crystallize in a type of columnar grain, where the grains are the finest in STZ and the coarsest in HAZ. In addition, the arc swing improves the microstructure homogeneity of the hardfacing layers by obviously lowering the tempering degree in HAZ while promoting the even distribution of the arc heat. Accordingly, the hardness of the SA-SAW bead overall increases and distributes more uniformly with a maximum difference of < 80 HV_0.5_ along the horizontal direction of the bead. This hardness difference in SA-SAW is accordingly decreased by ~38.5% compared to that of the SAW bead, further indicating the practicability of the new process.

## 1. Introduction

In the steel rolling process, the hot-rolled backup roll, as an essential component of the rolling mill, is the main consumption part [1]. This backup roll is operated at high temperatures and bears a larger load than the work roll, so its performance requirements are very high [2]. The backup roll is usually made of forged steel with high C and Cr and has the performances of high wear resistance and good antistrip [3]. However, after the backup roll serves under high load for a period of time, the roll surface is prone to spalling, making it unable to work properly. For the backup roll, because of its high technical requirements and difficulties in fabricating, the hardfacing technique is currently used for the roll surface repair [4,5,6].

Recently, many surface modification processes, such as laser cladding [7,8], plasma arc cladding [9,10], and submerged arc surfacing [11,12], have been used to fabricate thick cladding layers. In these processes, submerged arc surfacing was used to repair the roll surface [12] because of its easy operation, high deposition efficiency, and low welding cost. However, the submerged arc process actually leads to a corrugated-groove defect on the roll surface repaired due to the uneven hardness distribution in the hardfacing layer, affecting the usage of the roll. Therefore, it is necessary to explore a new low-cost process to solve this problem. Actually, arc heat distribution during the welding process is key to governing weld formation and the microstructure and mechanical properties of welding joints. To improve this distribution, several rotating/weaving arc processes were developed, such as the rotating arc [13], mechanically swing-arc [14], and magnetically oscillating arc [15]. The arc can regulate its heat and force distributions in a welding groove. Of these processes, the swing-arc is most attractive because of its good wire directivity and moving parameter controllability [16].

To overcome the drawbacks of the current processes, a novel swing-arc submerged arc welding (SA-SAW) process is proposed. This swing-arc regulates the transverse distribution of the arc heat in the welding pass to flatten the weld and form a hardfacing layer that smoothes the bottom, which is expected to improve the evenness of the microstructure and hardness. The effect of arc swing parameters on the hardfacing formation is investigated. Additionally, the characteristics of the microstructure and hardness of the hardfacing layers are analyzed. This aids in further understanding the SAW hardfacing process and promotes the application of the SAW process in backup roll repair.

## 2. Materials and Methods

### 2.1. Welding System

**Figure 1:**
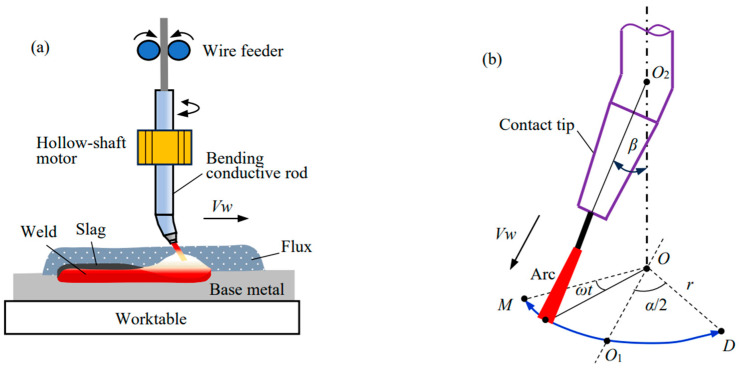
Schematic illustration of experiment setup: (**a**) System configuration; (**b**) Arc swing model.

Figure 1 illustrates the experiment setup for the hardfacing process. A welding supply (MZ-1250, Aotai, Jinan, China), which has a descending output characteristic and matches a wire feeder of a variable rate, was used for submerged arc hardfacing. Its positive and negative terminals are, respectively, connected to the swing-arc torch and base metal. The welding torch [17], including the hollow-shaft motor, bending conductive rod, and straight contact tip, adopts a conductive rod of bending angle *β* of 10° to circularly swing the arc at an angular velocity of *ω* relative to the torch central line *OO*_2_, the swing trace of which is indicated by the arc-shape line *MO*_1_*D* in Figure 1b. Accordingly, arc swing parameters contain swing angle *α*, swing radius *r*, and side-dwell time *t*, respectively, at the left and right limiting points *M* and *D* of the swing. This swing at several Hertz can effectively regulate the distribution of the heat and force of the arc across the weld. In the experiments, a worktable carries the base metal to move at the welding speed of *Vw*.

### 2.2. Materials and Welding Process

A wear-resistant flux-cored welding wire (THY-MD401-5, Daqiao, Tianjin, China) with a diameter of 4.0 mm was used for SAW hardfacing. Table 1 lists the chemical composition of the welding wire. The base metal was Q370qE steel with a dimension of 240 mm × 240 mm × 30 mm. Table 2 shows the welding experimental conditions. During surfacing, in order to avoid the quenching effect of the welding pass/layer, the inter-pass/layer temperature needs to be strictly controlled. Accordingly, the preheating and inter-pass/layer temperatures must be slightly higher than the martensite transformation temperature *Ms* of the weld metal. If these temperatures are lower than the value of *Ms*, a quenching effect occurs in the welded pass and thus leads to the unevenness of its microstructure and hardness. The value of *Ms* can be determined by the following equation [18]. According to Table 1 and Equation (1), the value of *Ms* was calculated as 314 °C. Actually, the dilution rate of weld metal lowers *Ms*, so the calculated value is a safe temperature to prevent the martensite transformation during hardfacing.

(1) $$Ms\left({}^{\circ}C\right)=512-453\left(\%C\right)-16.9\left(\%Ni\right)+15\left(\%Cr\right)-9.5\left(\%Mo\right)+217{\left(\%C\right)}^{2}-71.5\left(\%C\right)\left(\%Mn\right)-67.6\left(\%C\right)\left(\%Cr\right)$$

**Figure 2:**
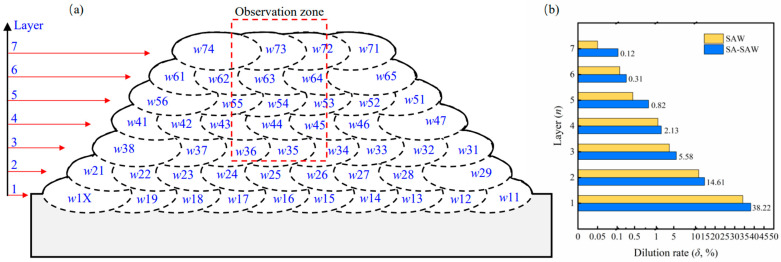
Hardfacing layers details for (**a**) schematic illustration: *w*11 represents the 1st pass in the 1st layer, *w*1Ⅹ denotes the 10th pass in the 1st layer, and so forth; (**b**) dilution rate of each welding layer.

On the other hand, the dilution rate *δ* is one of the key factors affecting the hardfacing performance. The rate *δ*_1_ of the first welding layer can be determined by the cross-sectional area ratio of the melted base metal to the weld [19], and the rate *δ_n_* of the *n*-th layer is approximately denoted as ${\delta_{1}}^{n}$. To obtain the welding layers of low dilution rate, a hardfacing bead of 7 layers was schemed, as shown in Figure 2a. The actual values of the rate are correspondingly calculated and indicated in Figure 2b. With the increasing number of layers, the rate rapidly decreases. In practice, several uppermost layers of low dilution rate are commonly employed as the serving layer after the roll is repaired. In our work, the dilution rate of the uppermost three layers is <1% (see Figure 2b), the layers of which exactly reflect the best wear-resistant properties. Thus, the three layers are selected as examples to be investigated in the observation zone. Additionally, this zone can demonstrate the cross-sectional macro morphology of the hardfacing bead by the upper 5 layers.

### 2.3. Microstructural Characterization

The microstructure of the bead was analyzed using an optical microscope (Olympus BXM53M, Tokyo, Japan). To observe the microstructure, specimens were prepared by mechanically polishing with #60~#2000 emery papers and then sequentially with 3.5 μm, 2.5 μm, and 1.5 μm WC suspensions. Finally, a chemical etching to emphasize the microstructure was performed with a Nital 8% solution for 20 s.

**Figure 3:**
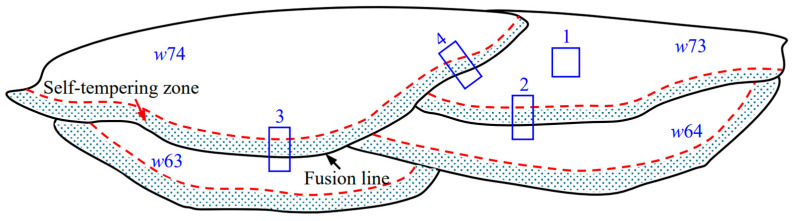
Schematic illustration of the neighboring two-pass welds in the 6th and 7th layers: Zone 1, 2, 3, and 4 indicate the microstructure analyzing areas.

Figure 3 illustrates a schematic diagram of the neighboring two-pass welds in the 6th and 7th layers, where Zone 1, 2, 3, and 4 indicate the microstructure analyzing areas in subsequent discussions. In the figure, the solid black line in the overlapping region between the welding passes/layers denotes the fusion line, and the narrow region surrounded by the red dotted line and the fusion line is the self-tempering zone (STZ).

### 2.4. Hardness Testing

**Figure 4:**
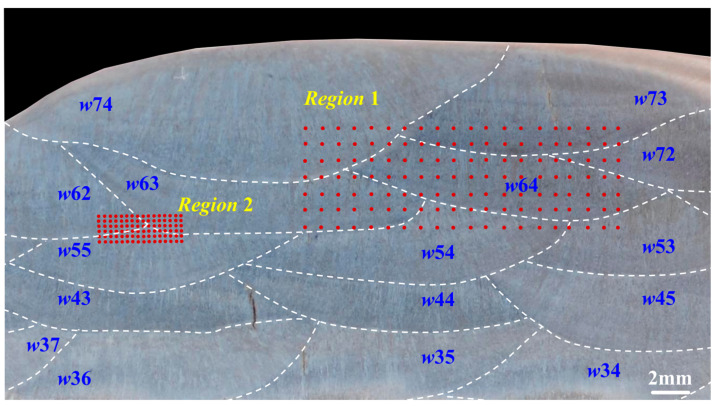
Cross-sectional macrograph example of the observation zone for the SA-SAW hardfacing layers: Red points indicate the hardness testing points; Region 1 and Region 2 denote the hardness testing areas with point-to-point intervals of 1 mm and 0.3 mm, respectively.

Figure 4 shows the cross-sectional macrograph in the observation zone for the hardness test. This picture covers the 3rd to 7th layers of the SA-SAW hardfacing bead. Region 1 was selected to reflect a hardness distribution of the 7th to 5th welding layers. It covers 20 points × 7 points, where the point-to-point interval is 1 mm. Region 2, which contains 16 points × 6 points with point-to-point intervals of 0.3 mm, was used to analyze the local hardness features. An automatic hardness tester (KB 30S FA BASIC, Hochdorf-Assenheim, Germany) was used to measure the Vickers hardness of the two regions at a load of 0.5 kgf for 10 s.

## 3. Results and Discussion

### 3.1. Hardfacing Formation

#### 3.1.1. Formation of Single-Pass Weld

**Figure 5:**
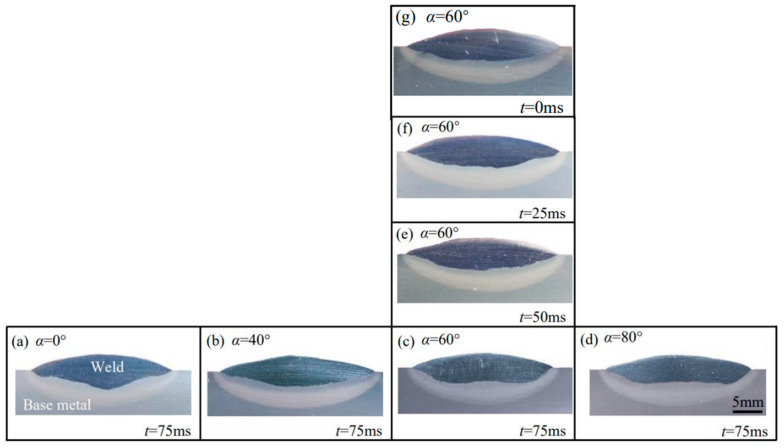
Cross-sectional macrographs of single-pass weld at the swing frequency of 3 Hz: (**a**–**d**) Effect of swing angle *α*, (**c**,**e**–**g**) Effect of side-dwell time *t*.

**Figure 6:**
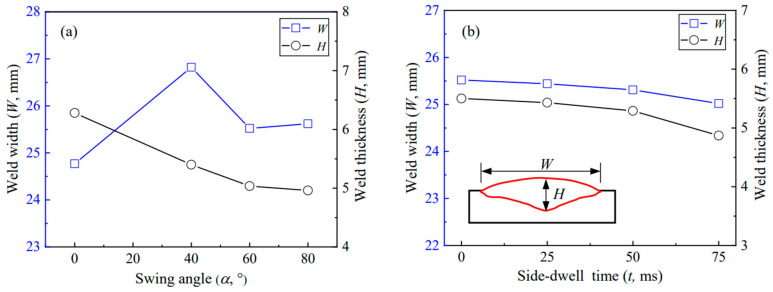
Effect of arc swing parameters on weld width and weld thickness at the swing frequency of 3 Hz: (**a**) Effect of arc swing angle *α*, (**b**) Effect of side-dwell time *t*.

Figure 5a–g gives the cross-sectional macrographs of a single-pass weld at different swing parameters, where the swing frequency is 3 Hz. With increasing the swing angle *α* from 0° to 80°, the distributions of the arc energy and arc force on the sides of the bead increase, while those in the middle region of the bead decrease. Additionally, the acceleration and deceleration during the arc swing raises the arc energy near the sides of the bead. Consequently, as shown in Figure 5a–d, the heat and force of the arc gradually distribute evenly across the weld, and thus the bottom of the weld becomes flat. Accordingly, the weld thickness decreases. Simultaneously, the weld width is greater at *α* ≥ 40° than at *α* = 0° and decreases with the reduction of *α* from 40° to 80°, as shown in Figure 6a. This reduction is because the increased penetration at both sides of the weld causes the arc to submerge during dwelling. A similar effect was reported in the SAW by arc weaving [20].

As the side-dwell time increases from 0 ms to 75 ms, the more heat and force of the arc distribute at both sides, and accordingly, the weld goes thin while the penetration at both sides increases, as shown in Figure 5c,e–g. Additionally, also due to the submergence of the arc at both sides, the arc shrinks, resulting in a decrease in the weld, as shown in Figure 6b. The results show that the flat weld of sound formation can be obtained with the arc swing parameters of *α* = 60° and *t* = 75 ms.

#### 3.1.2. Formation of Overlap Weld

**Figure 7:**
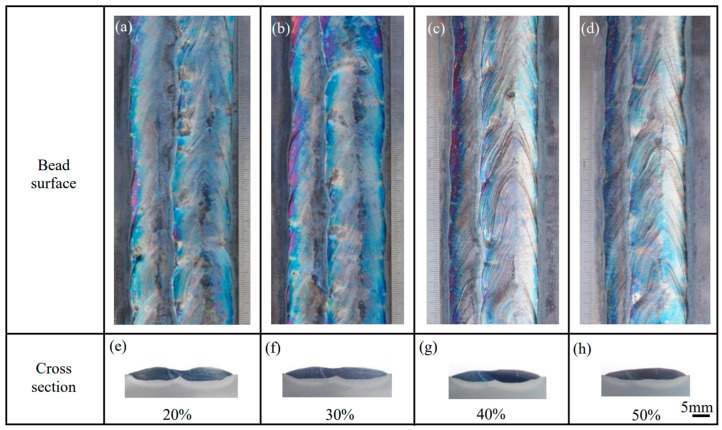
Macrophotographs of two-pass overlap welding bead at different overlap ratios: (**a**–**d**) Surface morphology, (**e**–**h**) Cross-section morphology.

To investigate the effect of the overlap ratio on the hardfacing bead, a number of two-pass overlap welding experiments are carried out at the swing parameters determined above. Figure 7 shows the two-pass overlap welding bead at different overlap ratios. When the ratio increases, the overlapping place gradually becomes flat at the bottom of the weld. The two-pass weld is the flattest at a ratio of 50%, as shown in Figure 7h. Simultaneously, Figure 7a–d demonstrates that the weld surfaces are becoming smooth.

#### 3.1.3. Formation of Multilayer Hardfacing

**Figure 8:**
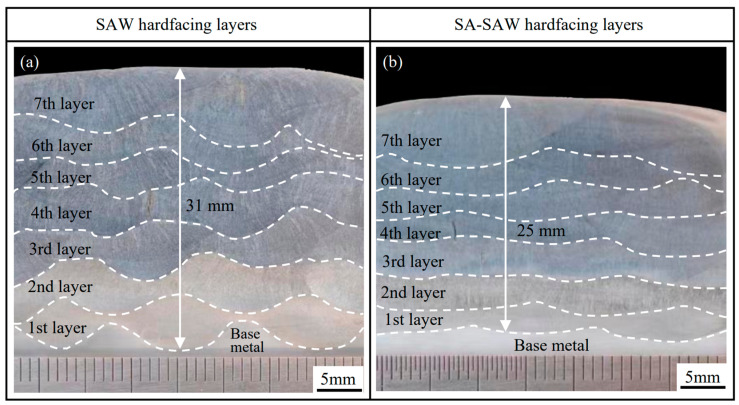
Macrophotographs of the cross-section of hardfacing layers: (**a**) Hardfacing by SAW, (**b**) Hardfacing by SA-SAW.

Figure 8 shows the cross-sectional macrophotographs in the middle section of the SAW and SA-SAW hardfacing beads. Here, the dotted white lines indicate the positions of the fusion lines from the 1st to 7th hardfacing layers. The arc swing flattens the welding pass, and accordingly, each hardfacing layer of the SA-SAW bead becomes thin. As a result, the overall thickness of the SA-SAW hardfacing layers is clearly less than that of the SAW hardfacing layers. In the SAW bead, the heat and force of the arc concentrate in the middle of each welding pass, which causes a big penetration in the center of each pass. Consequently, the fusion line exhibits a wavy shape, as shown in Figure 8a. Comparatively, the fusion line becomes smooth in the SA-SAW hardfacing layers due to the swing effect of the arc, as shown in Figure 8b. Moreover, with the increase in the number of layers, the dilution rate decreases, which leads to the change of the layer color from light to dark. Obviously, the layer color becomes consistent.

### 3.2. Microstructure Analysis

#### 3.2.1. Microstructure of the Not-Heat-Affected Weld Metal

**Figure 9:**
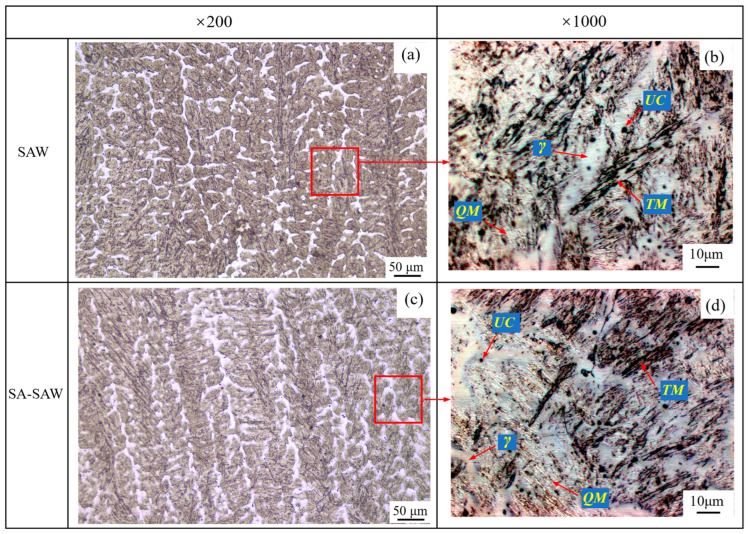
Optical cross-sectional micrographs of the not-heat-affected weld metal in Zone 1 (Figure 3) in the *w*73 welding pass: (**a**,**b**) SAW hardfacing layers, (**c**,**d**) SA-SAW hardfacing layers, *γ*-Austenite, *QM*-Quenched martensite, *TM*-Tempered martensite, *UC*-Undissolved carbide.

Figure 9 shows the microstructure in the cross-section of the weld metal not-heat-affected, which corresponds to Zone 1 (in pass *w*73) in Figure 3. On the whole, the weld metal crystallizes in a columnar grain type, but the grain is finer in SA-SAW weld, as shown in Figure 9a,c. The latter case is because the arc swing mechanically stirs the molten pool and promotes the uniform distribution of the arc heat across the weld. Additionally, as shown in Figure 9b,d, the microstructures of this zone mainly consist of white quenched martensite (*QM*), black–gray tempered martensite (*TM*), white austenite (*γ*), and a small amount of black undissolved carbide (*UC*) particles. The occurrence of more austenite results from the high contents of manganese (see Table 1), where the manganese is actually used as an austenite stabilizer. This austenite was also observed in the martensitic hardfaced SAW cladding by Buntoeng, S. et al. [21]. Particularly, the tempered martensite suggests that self-tempering indeed exists in the not-heat-affected weld metal zone.

#### 3.2.2. Microstructure of the Overlapping Zone between Layers

**Figure 10:**
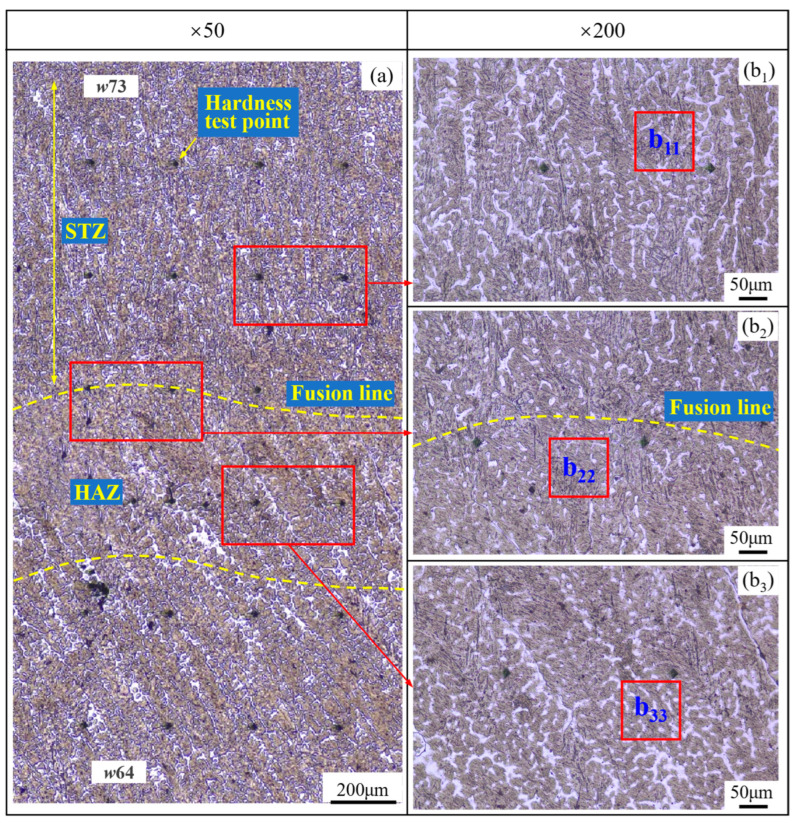
Microstructure in the overlapping Zone 2 (Figure 3) between welding passes *w*73 and *w*64 in the neighboring SAW layers: (**a**) Morphology at low magnification, (**b_1_**–**b_3_**) Magnified morphology.

**Figure 11:**
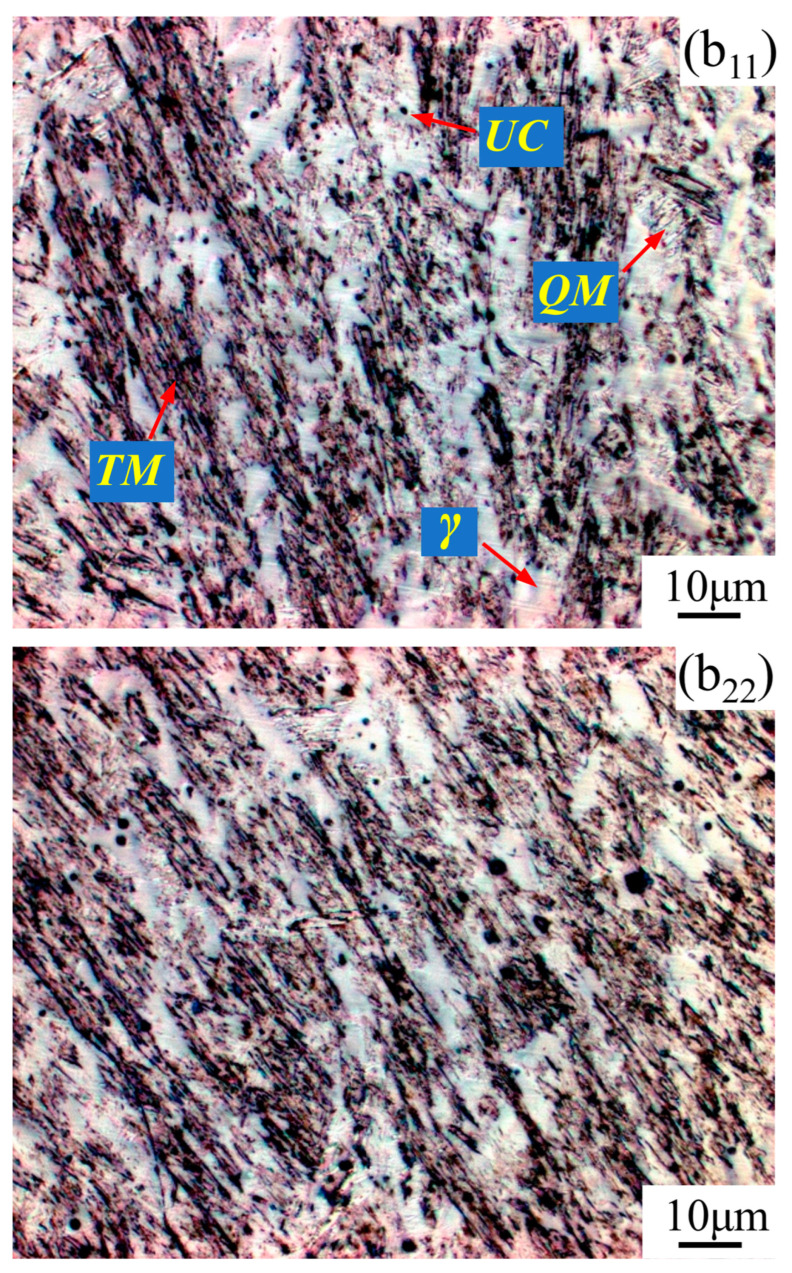

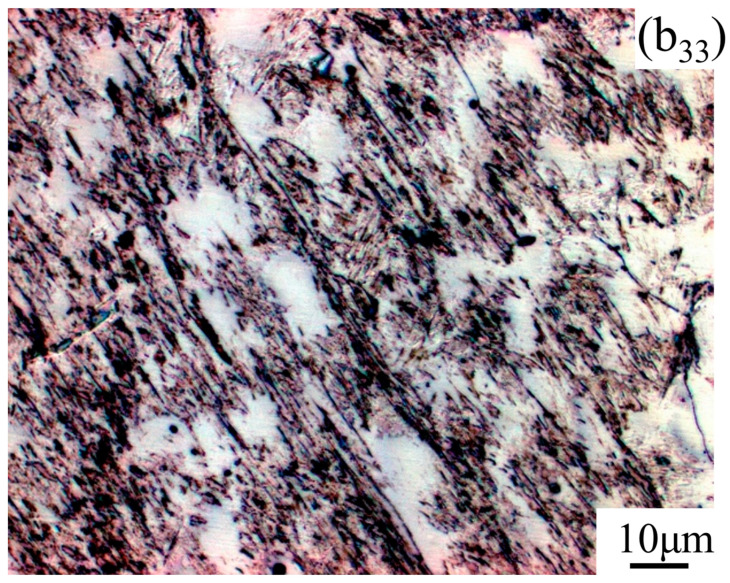
SAW layer microstructure in the observation zones (**b_11_**–**b_33_**) (Figure 10b_1_–b_3_): magnified with ×1000.

**Figure 12:**
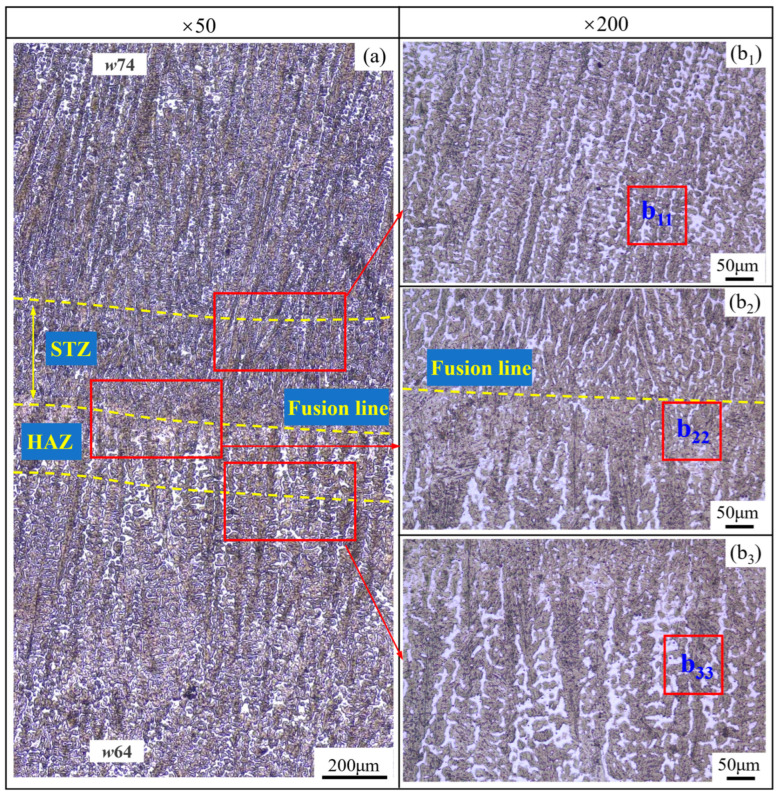
Microstructure in the overlapping Zone 3 (Figure 3) between welding passes *w*74 and *w*63 in the neighboring SA-SAW layers: (**a**) Morphology at low magnification, (**b_1_**–**b_3_**) Magnified morphology.

Figure 10 and Figure 11 show the microstructure in the overlapping Zone 2 (Figure 3) between welding passes *w*73 and *w*64 in the neighboring SAW layers, where an obvious HAZ occurs at the top of *w*64. As shown in Figure 10a, the microstructure in the bottom of pass *w*73 seems refined, which is considered to be caused by the self-tempering effect. Self-tempering occurs due to the migration of carbon atoms in the formed martensite [22,23] during the cooling process of the medium-alloyed steel weld with a higher value of *Ms* [24]. In the self-tempering, carbon atoms in a high-energy state in martensite migrate and gather near the lower-energy dislocations and the martensite boundaries and finally precipitate in the form of carbides. This precipitated phase produces a “pinning effect” at the grain boundaries to hinder grain growth, which is the main cause of grain refinement [25]. In the case of SA-SAW, the self-tempering zone narrows due to a larger temperature gradient during the cooling, as shown in Figure 12a.

**Figure 13:**
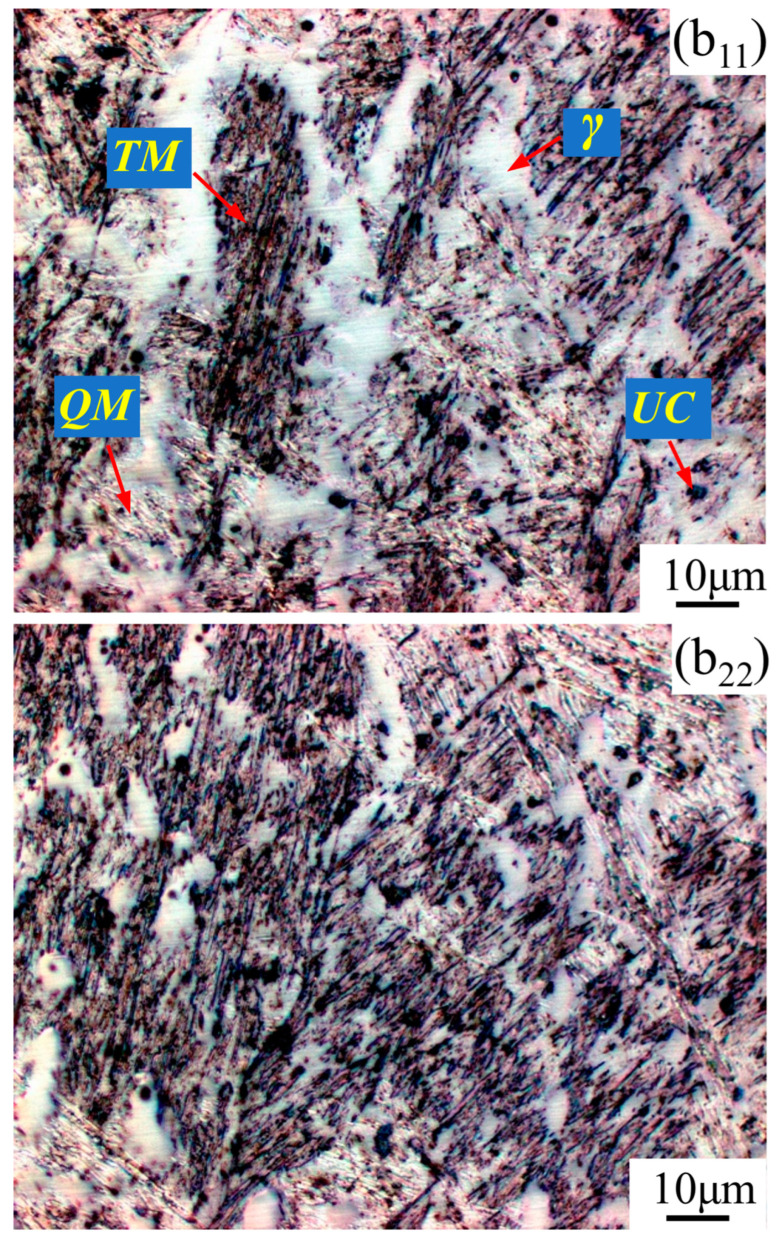

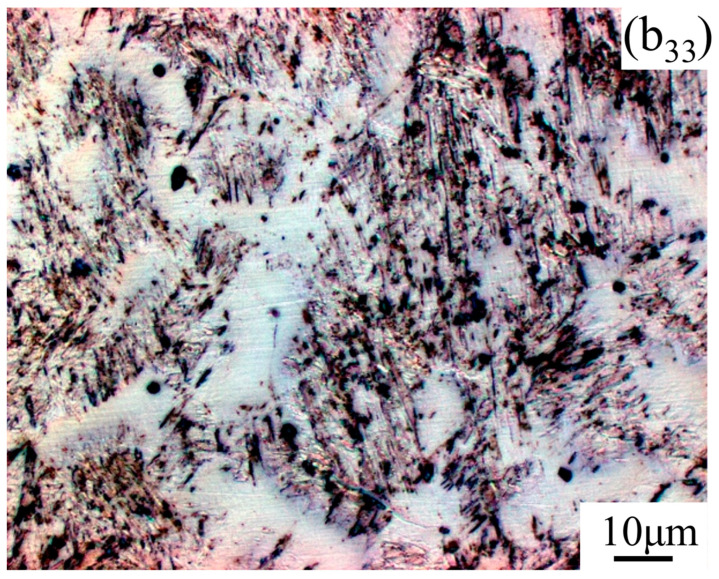
SA-SAW layer microstructure in the observation zones (**b_11_**–**b_33_**) (Figure 12b_1_–b_3_): magnified with × 1000.

From the microstructure, as shown in Figure 11b_11_,b_22_ and Figure 13b_11_,b_22_, four types of structures occur in the STZ and HAZ of the two overlapping zones, the microstructures of which include the tempered martensite (*TM*), the quenched martensite (*QM*), the austenite (*γ*), and the undissolved carbide (*UC*). Compared to the SAW overlapping zone in Figure 10a, HAZ obviously narrows in the SA-SAW overlapping zone in Figure 12a since the uniform distribution of the swing-arc heat weakens the heat effect of the later-layer welding pass (*w*74) on the former-layer pass (*w*63). In HAZ, the microstructure is clearly coarse columnar grain structure (see Figure 10b_3_ and Figure 12b_3_). Additionally, in the case of SAW, the black–gray tempered martensite increases while the austenite decreases, as shown in Figure 11b_33_ and Figure 13b_33_, which suggests that the degree of tempering in this HAZ is high.

In STZ, as shown in Figure 10b_1_ and Figure 12b_1_, the weld solidifies in a similar columnar grain type to that in Figure 9a,c, but the grain is refined. In the case of SAW, the extent of the self-tempering is high due to the concentrated distribution of the arc heat, which is demonstrated by the microstructure feature of less austenite and more tempered martensite in Figure 11b_11_. Inversely, the austenite increases, and the tempered martensite decreases for SA-SAW in Figure 13b_11_, which indicates a low degree of self-tempering. As seen from the top region of HAZ beneath the fusion line, furthermore, the austenite decreases clearly, and the tempered martensite bundles go denser compared to the other regions, as shown in Figure 11b_22_ and Figure 13b_22_, which implies tempering to the highest extent.

From the above characteristics of the various regions, a softening may occur in HAZ with the features of tempering, of which the softest zone should be the top of HAZ due to the existence of the most obvious tempering. Inversely, the hardness should rise in STZ because of the grain refining caused by the self-tempering. Compared to the case of SAW, the SA-SAW process narrows the regions of HAZ and STZ while reducing the extent of the tempering and the self-tempering, which lowers the differences in the microstructure and properties between the hardfacing layers.

#### 3.2.3. Microstructure of the Overlapping Zone between Passes

**Figure 14:**
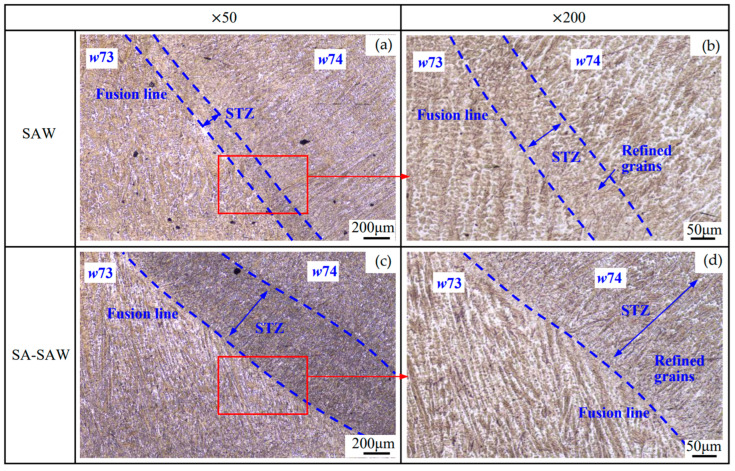
Microstructure in the overlapping Zone 4 (Figure 3) between welding passes *w*73 and *w*74: (**a**,**b**) in SAW, (**c**,**d**) in SA-SAW.

Figure 14 shows the microstructure in the overlapping Zone 4 (Figure 3) between welding passes *w*73 and *w*74. Similar to the case of the above overlapping zone between layers, STZ also appears to be the overlapping zone between the passes. Compared to SAW, the self-tempering is more significant in SA-SAW because of the stronger heat effect of the swing-arc on the two sides of the welding pass, resulting in a wider STZ, as shown in Figure 14a,c. Apart from STZ, the features of columnar crystal are exhibited clearly in both processes. In addition, it can be seen from Figure 14b,d that the grains are refined in STZ owing to the strong self-tempering in SA-SAW and become fine in the other regions of the overlapping zone due to the stirring effect of the swing-arc. Moreover, the arc heat always distributes less on both sides than in the middle of the welding pass, which probably causes an obvious HAZ to disappear in this zone.

According to the above features of the microstructure in this zone, no softening occurs due to the lack of the obvious HAZ in the overlapping zone between passes. On the whole, compared to SAW, the SA-SAW process refines the grains in the above three regions of the not-heat-affected and the two overlapping zones and thus should produce a hardfacing bead of higher hardness.

### 3.3. Hardness Properties

#### 3.3.1. Overall Hardness of Hardfacing Layers

**Figure 15:**
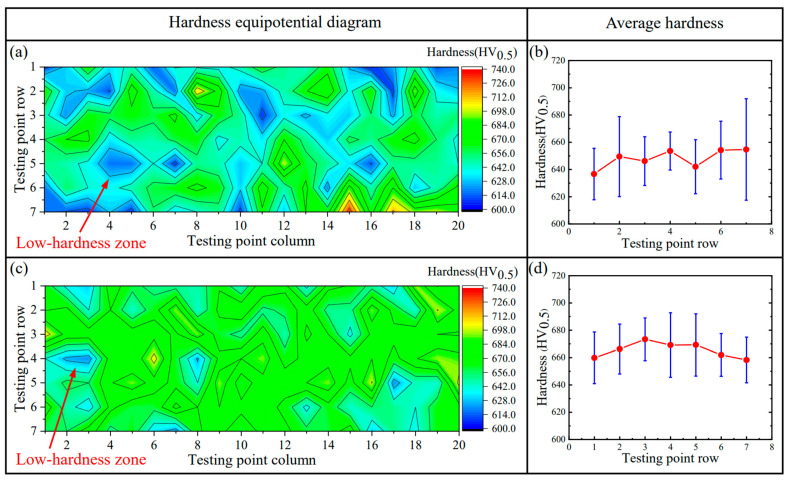
Hardness distribution of the hardfacing layers in the red-point Region 1 in Figure 4: (**a**,**b**) SAW, (**c**,**d**) SA-SAW.

Figure 15a,c is the equipotential diagram of the hardness and intuitively reflects the hardness distribution of the hardfacing layers in the red-point Region 1 in Figure 4. This diagram is estimated from the point matrix of 20 points × 7 points in Region 1 by utilizing the Origin software (2021 version). The color bar indicates the hardness value in the diagram. In SAW (see Figure 15a), the more blue and red areas in the diagram, respectively reflecting the low and high hardnesses, appear dispersedly, which suggests an ununiform distribution of the hardness. This is actually induced by the large difference mentioned above in the microstructure of the hardfacing layers. In SA-SAW (see Figure 15c), both the red and blue areas are few, implying a relatively even distribution of the hardness, which corresponds to the microstructure features (see Section 3.2) of its hardfacing layers.

In addition, Figure 15b,d indicates the average value and standard error of the hardness along the row of the test points, respectively, for the SAW and SA-SAW processes. Compared to the SAW process, the average hardness of the SA-SAW hardfacing layers increases by ~20 HV_0.5_, while the standard error clearly decreases. That is to say, this new process can fabricate the hardfacing layers of the higher and more uniform distribution of the hardness.

#### 3.3.2. Local Hardness of Crossing Region among the Passes and Layers

**Figure 16:**
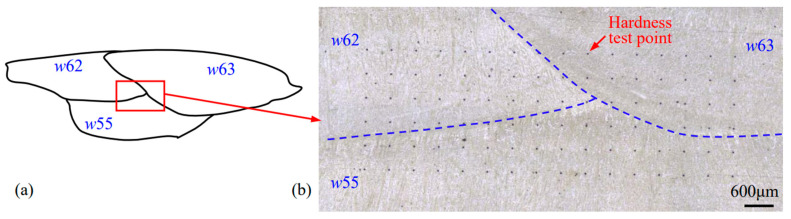
Diagram of the hardness testing Region 2 covering the three welding passes of *w*62, *w*63, and *w*55: (**a**) Illustration, (**b**) Micrograph of the SA-SAW hardfacing layers.

**Figure 17:**
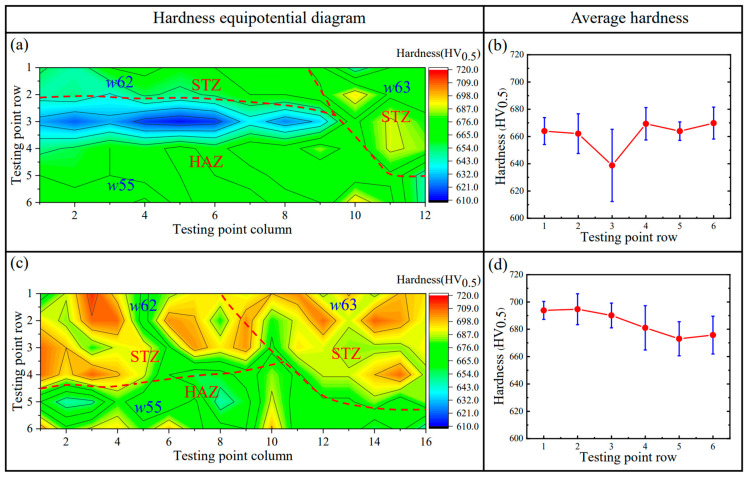
Hardness distribution of the red-point Region 2 (Figure 4) covering the three welding passes of *w*62, *w*63, and *w*55: (**a**,**b**) SAW, (**c**,**d**) SA-SAW. The two red dotted lines denote the fusion lines and divide the equipotential diagram into 3 parts, which correspond to an STZ in *w*62, an STZ in *w*63, and a HAZ in *w*55.

To explore the forming cause of the low-hardness zones in Figure 15, another hardness testing was carried out in Region 2 (see Figure 4) across the three welding passes (*w*62, *w*63, and *w*55) in the 6th and 5th layers. Figure 16 accordingly gives an example of an actual micrograph of the hardness test points in Region 2 for SA-SAW. Figure 17a,c and Figure 17b,d are the equipotential and average value diagrams of the hardness, respectively. In Figure 17a, a low-hardness zone with a blue color appears near the 3rd row of the testing points, the row of which is just in HAZ between the 6th and 5th layers. Based on the above microstructure analysis in Section 3.2.2, a tempering occurs in the HAZ. It is judged that the tempering in HAZ is the cause of the softening in the hardfacing layers.

For the SA-SAW process, a low-hardness zone also appears near the 5th row in Figure 17c, the row of which is actually in HAZ. In addition, the yellow areas with higher hardness cover from the 1st to 4th rows in Figure 17c, where most of the testing points in these rows are examined to be exactly located in STZ according to the actual positions of the points. This verifies that the self-tempering raises the layer’s hardness. Because of the grain refining effect of the swing-arc, however, the hardness values in this STZ are greater than those in another STZ of SAW layers, the later STZ, which is situated beyond HAZ in Figure 17a.

Nonetheless, the fluctuations in the average hardness in SA-SAW are relatively small, as reflected in Figure 17d. Actually, the average hardness difference among all six rows is below 30 HV_0.5_, the value of which is clearly lower than the difference value of 60 HV_0.5_ in SAW in Figure 17b. Particularly, it can be seen that the maximum difference of the hardness in one row significantly becomes low while comparing the error bar (standard error) in the 4th row of the SA-SAW hardness points to that in the 3rd row of the SAW ones, as shown in Figure 17b,d. The results demonstrate that the new process can avoid the occurrence of the low-hardness zone in the overlapping regions of the hardfacing layers and accordingly improves the hardness distribution.

**Figure 18:**
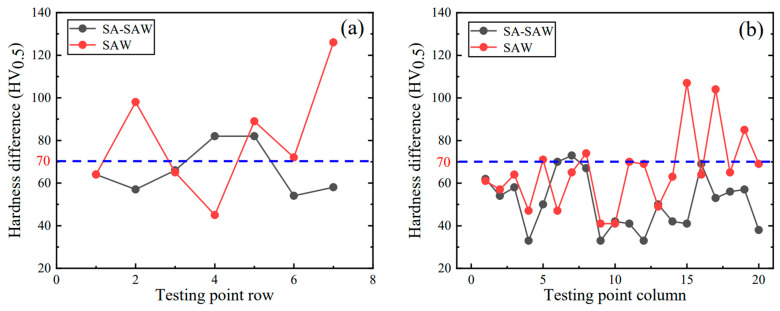
Hardness difference distribution of the hardfacing layers in the red-point Region 1 in Figure 4: (**a**) along the testing-point row, (**b**) along the testing-point column.

Finally, the difference between the maximum and minimum of the hardfacing hardness is counted for each row and column of the testing points in Region 1, respectively. The results are indicated in Figure 18. In SA-SAW, the maximum difference of the hardness is ~80 HV_0.5_ in the rows of the testing points, while the hardness difference in SAW approaches 130 HV_0.5_, as shown in Figure 18a. From the column of the testing points, on the other hand, the maximum hardness difference in SA-SAW is around 70 HV_0.5_, while the hardness difference in SAW rises up to ~110 HV_0.5_, as shown in Figure 18b. This hardness difference in SA-SAW is decreased by ~38.5% and ~36.4%, respectively, for the statistics along the rows and columns of the testing points relative to that of the SAW bead. Clearly, the hardness difference in SA-SAW is smaller and approaches the expected value of 3.0 HRC (approximately 70 HV_0.5_) by industrial application, indicating the practicability of this new process.

## 4. Conclusions

1. A new SA-SAW process fabricates a hardfacing bead with a uniform and thin interlayer relative to SAW since the arc swing effectively regulates the transverse distribution of the arc heat and the arc force in the welding layer. Accordingly, the arc swing parameters were optimally selected as the swing frequency of 3 Hz, the swing angle of 60°, the side-dwell time of 75 ms, and the overlap ratio of 50%.
2. Each interlayer hardfacing pass clearly includes the self-tempering zone (STZ) in its bottom and the heat-affected zone (HAZ) in its upper region, while each cap pass contains two self-tempering zones, respectively, in its bottom and its side. In all the zones, including STZ, HAZ, and not-heat-affected zone in the pass, both SA-SAW and SAW passes crystallize in a type of columnar grain, where the grains are the finest in STZ and the coarsest in HAZ. Additionally, each zone covers four types of microstructures, i.e., quenched martensite, tempered martensite, austenite, and precipitated carbides.
3. The arc swing can improve the microstructure homogeneity of the hardfacing layers by obviously lowering the tempering degree in HAZ while promoting the even distribution of the arc heat. Accordingly, the hardness of the SA-SAW bead overall increases because of the grains refined by the arc swing and distribute more uniformly with a maximum difference of < 80 HV_0.5_ along the horizontal direction of the bead due to an obvious rise of the hardness in HAZ. This hardness difference in SA-SAW is decreased by ~38.5% compared to that in SAW, further indicating the practicability of the new process.

## Figures and Tables

**Table 1:** Chemical composition of the welding wire (wt.%).

C	Mn	Si	V	Cr	Ni	Cu	Mo	W	Co	Al
0.298	1.845	0.47	0.557	6.007	0.039	0.032	0.839	1.577	0.015	0.015

**Table 2:** Welding experimental conditions.

Parameter/Type	Value/Mode
Average welding current (A)	500~520
Average welding voltage (V)	30~31
Welding speed (mm/min)	300
Torch standoff height (mm)	32
Interlayer temperature (°C)	320
Number of hardfacing layers	7
Flux	A5.39M (AWS)

## Data Availability

Data are contained within the article.
